# Particle-Filled Emulsion Drops Show Flow-Induced Partial Coalescence, but Only Transiently

**DOI:** 10.1021/acs.iecr.5c02170

**Published:** 2025-11-10

**Authors:** Jovina Vaswani, Sachin S. Velankar

**Affiliations:** † Department of Chemical Engineering, 6614 University of Pittsburgh, Pittsburgh, Pennsylvania 15261, United States; ‡ Department of Mechanical Engineering and Material Science, University of Pittsburgh, Pittsburgh, Pennsylvania 15261, United States

## Abstract

Partial coalescence refers to a process where two or more droplets come into contact and merge, but do not recover spherical shape. We conduct a flow-visualization study of the shear flow-induced partial coalescence of an emulsion of particle-filled drops. Experiments are conducted with poly­(ethylene oxide) drops dispersed in polyisobutylene. The drops are filled to over 50 vol % with spherical silica particles. Partial coalescence is attributable to the solid-like behavior induced by the particles inside the drops and possibly at the interface. After subjecting the emulsions to high-rate shear, the drops adopt slightly nonspherical shapes and retain them even under quiescent conditions. Subsequent shearing at lower rate causes these drops to partially coalesce into highly irregular drop shapes, and particles promote this coalescence process. But with continued shearing, the highly irregular drops gradually become rounded and approximately spherical. The reversion to rounded shape is faster at higher shear rate and at high drop loading. In contrast, at low rates or low drop loadings, drops can sustain grossly irregular shapes even when sheared for hundreds of strain units. This gradual reversion to sphericity is not driven by capillarity. Instead, we propose that the chief mechanism is that when irregularly shaped drops collide, the viscous stress in their near-contact region induces localized yielding and particle rearrangements. Thus, repeated drop collisions gradually smoothen the drops toward sphericity.

## Graphical abstract

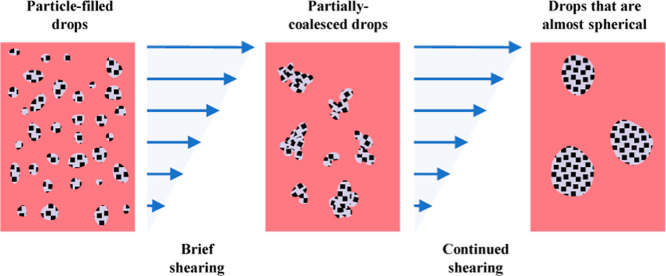

## 1 Introduction

When two drops coalesce, the desire to minimize surface energy favors an eventual drop shape that is perfectly spherical. However, when liquid drops containing solid particles come into contact, coalescence can be arrested at an intermediate stage, where the drops merge into a single body, but do not recover a spherical shape. In effect, the particles endow the drops with solid-like mechanical properties that resist the driving force of surface energy minimization. The food science community, in particular, has extensively studied this phenomenon of arrest, using the term “partial coalescence”.^1–4^ The case where volume elasticity (i.e., solid-like behavior throughout the volume of the dispersed phase) leads to arrested structures is important to structured food emulsions such as ice cream and whipped cream.^5^ The milk fat emulsion can be churned to butter only when part of the fat inside the emulsion droplets has crystallized and developed an elastic stress.^6^ Spicer and co-workers^7,8^ have used the apt term “drops with an endoskeleton” to describe this situations. Incidentally, irregular drop shapes can often appear due to irreversible adsorption of particles or other surface-active species at the interface. Such particle-stabilized emulsions, called Pickering emulsions, often show nonspherical drop shapes and refs 9–13 show especially clear examples of the same. Such cases of “drops with an exoskeleton” where the solid-like behavior appears at the interface and not throughout the volume of the drop, are not discussed here.

Some previous studies have examined partial coalescence under quiescent conditions, i.e. in the absence of external flow.^14–17^ Pawar et al. used micromanipulation stages and microcapillaries to grasp and move oil droplets into contact.^14^ These oil droplets contained elongated wax crystal particles. Once contact between the drops was established, the extent of coalescence was determined by the strength of the internal particle network. With a weak network, capillary forces dominated, and drops were found to coalesce completely into a spherical shape. With a strong crystal network, drops remained in contact with each other without merging to any significant degree. At intermediate network strength, however, partial coalescence into arrested dumbbell shapes was noted, indicative of a balance between elastic and capillary forces. The degree of coalescence was quantified across various parameters including temperature, volume fraction of wax, droplet radius, and polydispersity. These studies under quiescent conditions provide insights into the determination of a globule shape by a balance between elastic and surface energy.

However, during manufacture of food emulsions or cosmetic products, coalescence is generally induced by flow encountered during mixing or processing.^18^ Many cosmetic products are oil-in-water emulsions containing semicrystalline waxes, and the texture of such materials is influenced by the extent of shear-induced partial coalescence.^19,20^ Shear-induced partial coalescence of fat drops in partially crystalline oil-in-water emulsions has been the subject of ongoing research.^2,21,22^ As per a mechanism proposed over 40 years ago,^6^ van der Waals attraction induces an internal aggregation of the fat crystals within oil drops, that leads to the formation of a crystal network with solid-like properties. Under flow conditions, two drops with a crystal network may approach each other and collide. If sufficient liquid oil is available in the drops, it begins to flow around the crystal network reinforcing the link between the two drops.^23^ Complete coalescence, i.e. reversion to spherical shape, is prevented by the solid-like behavior of each drop.

Partial coalescence has also been noted in systems very different from those typical in the food industry, viz. blends of two immiscible polymers where particles selectively fill one of the polymer phases. Compared to the emulsions described in the previous paragraphs, these polymeric systems have a much higher viscosity, and the particles are not fat or wax crystals but instead materials such as silica, carbon black, titanium oxide, etc.^24–34^ Upon melt-blending such three component mixtures, the particle-filled phase often appears irregularly shaped.^35^ Our group examined this in detail in blends where spherical silica particles or fumed silica particles selectively fill one phase.^35–39^ Partially coalesced aggregates of the particle-filled phase appeared when that phase had solid-like rheology. These arrested structures were not observed for the same polymer blends without added particles since in that case, the corresponding phase had liquid-like rheology.^38^ An especially interesting aspect of this and previous research on polymer blends is that under some conditions, the partially coalesced aggregates reach a percolation threshold, upon which a bicontinuous morphology appears where the particle-filled phase as well as the particle-devoid phase are both spatially percolating. While such bicontinuous phases can appear even without particles, the composition range over which they appear, and their stability, are both greatly enhanced by particles.^38^ The ease of realizing bicontinuous structures by simply mixing together three species is potentially useful for applications such as membranes, filters, or tissue-growth scaffolds where mechanical robustness must be combined with a high permeability.

All these previous studies only examined the morphology resulting *after* some specified mixing operation. The morphological evolution *during* mixing remains unknown. A simple “thought experiment” can illustrate the issues involved. Consider a dispersion of particle-filled drops with a solid-like rheology, initially approximately spherical, subjected to simple shear flow. We anticipate that the applied flow drives collisions between the drops. If the drops are sufficiently solid-like, they may behave like rigid objects and retain their shapes under flow. If, however, they are capable of coalescence, this would create nonspherical partially coalesced drops. But continued flow would subject these irregular drops to further collisions, leading to even larger drops. Such growth cannot continue indefinitely: the irregular drops must either become sufficiently large to span the size of the entire system, and/or they must deform and reorganize, and/or they must reach a steady state where drop aggregation and rupture balance each other. This thought experiment may be extended to the case when particles can adsorb at the interface. As the initially spherical drops coalesce, the interface must become fully covered with particles. Once the interface is covered, either the drop size must stop growing, or the dispersed phase must become nonspherical, or the particles must start desorbing from the interface. Clearly then, even in these simple thought experiments starting with spherical drops in shear flow, the evolution of morphology with time is unknown, and cannot be gauged by morphological examination under quiescent conditions *after* cessation of flow. This paper examines such morphological evolution as a function of time in a model system comprising a dilute dispersion of drops that are highly filled with spherical particles. The drops adopt slightly irregular shape after a high rate shear, and upon reducing the shear rate, the particles promote partial coalescence into highly irregular shapes. We show that continued flow induces compaction, which may eventually approach an approximately spherical shape, i.e. complete coalescence. The effect of applied shear rate and volume fraction of the drops on the morphological evolution has been studied experimentally. To our knowledge, this is the first study on the time-dependent growth and subsequent shape-relaxation of partially coalesced drops due to applied flow.

## 2 Experiments

### 2.1 Materials

The ternary blend consisted of poly­(ethylene oxide) (PEO, η = 0.28 Pa·s at 80 °C), polyisobutylene (PIB, η = 10.8 Pa·s at 80 °C) and spherical silica particles (SP, diameter roughly 2–3 μm). PIB was the continuous phase in all the samples discussed in this paper. PEO is semicrystalline at room temperature, and its melting point is close to 60 °C, whereas PIB is liquid. The viscosity ratio of the particle-free PEO drops to the PIB continuous phase is 0.026. As compared to some of our previous research,^36–38^ the PIB is identical, the silica particles are from the same vendor but a different batch, whereas PEO has a lower viscosity. Electron microscopy images of similar particles were published previously.^36^ An optical microscopy image of the particles and the particle size distribution provided by the supplier are both shown in the Electronic Supporting Information (Figure S1). The densities of the three phases, which are used to convert from mass fraction to volume fraction, are listed in Table 1. We were unable to measure the interfacial tension at 80 °C for the exact pair of PEO and PIB. However, interfacial tension between PEO and PIB was measured for a higher molecular weight of PIB (BASF Oppanol B10) and found to be 7.5 mN/m at 80 °C. In much of the Results section, drop sizes range from several microns (although some drops may be even smaller) up to roughly 100 μm, and are subjected to a shear rate of 5 s^–1^. This gives a capillary number range from about 0.03 to about 0.35. Occasional drops are even larger, and would have a correspondingly higher capillary number. The particles have a strong preference to be wetted by PEO, and the images below will show that the particles are entirely or predominantly contained within the PEO drops.

**Table 1:** Materials Used

Materials	Supplier	Molecular weight g/mol	Viscosity (80 °C)	Density (g/mL)
poly(ethylene oxide) (PEO)	BASF	4000 (from manufacturer)	0.28 Pa·s	1.1
polyisobutylene (PIB)	Soltex	2400 (from manufacturer)	10.8 Pa·s	0.908
silica particles (SP)	Industrial Powders			2 (quoted by the manufacturer)

### 2.2 Sample Preparation

10% PEO and 90% PIB were hand-blended with a spatula in an aluminum dish at 80 °C. The resulting PEO-in-PIB “masterbatch” was transferred to a plastic Petri dish and kept at 10 °C for at least 30 min to complete crystallization of the PEO. Ternary samples of the desired composition were then prepared by blending appropriate quantities of this masterbatch, pure PIB, and silica particles. The blending was performed at room temperature. This mixing procedure, dubbed “cold mixing” is intended to ensure that all samples have the same size distribution of PEO drops^40^—since PEO drops are solidified before the mixing step, their size does not vary from sample to sample, and they are not lost to wetting surfaces such as the spatula or the mixing dish. Accordingly, the as-prepared sample is a suspension with solid silica particles and solidified PEO drops suspended in PIB. Once prepared, each sample was placed overnight in a vacuum chamber at room temperature to degas before loading into a rheometer at 80 °C. It is only after melting in the rheometer that the solidified PEO drops melt and wet the silica particles to form a combined dispersed phase.

The ternary composition is defined by the volume fraction of silica particles ϕ_SP_, the wetting phase (PEO) volume fraction ϕ_PEO_, and the volume fraction of PIB, ϕ_PIB_ = 1 – ϕ_PEO_ – ϕ_SP_. The ratio $\varrho =\frac{\phi_{PEO}}{\phi_{SP}}$ was kept fixed at 0.77. Accordingly, the volume fraction of the particles within the (particles + PEO) combined phase is ϕ_combined_= (1 + ϱ)^−1^ = 0.565.

### 2.3 Flow Visualization

Visualization experiments were conducted on an Anton Paar rheometer MCR 302 in a 25 mm parallel plate geometry equipped with flow visualization capabilities. The area that was visualized was approximately 5 mm away from the center and left unchanged during all the experiments. The sample was viewed in the velocity–vorticity plane. All the experiments were conducted at 80 °C to ensure that PEO was well above its melting temperature. The gap between the plates was set to 200 μm.

The sample was preconditioned as per Figure 1: sheared at 200 s^–1^ for 2000 strain units and then sheared at 70 s^–1^ for 4000 strain units. Following preconditioning, shearing was continued at the desired rate (15 s^–1^, 5 s^–1^ or 1.5 s^–1^). The shear was stopped at specific strain intervals and images were recorded under static conditions; imaging under flow gave unacceptable degrees of motion blur.

**Figure 1:**
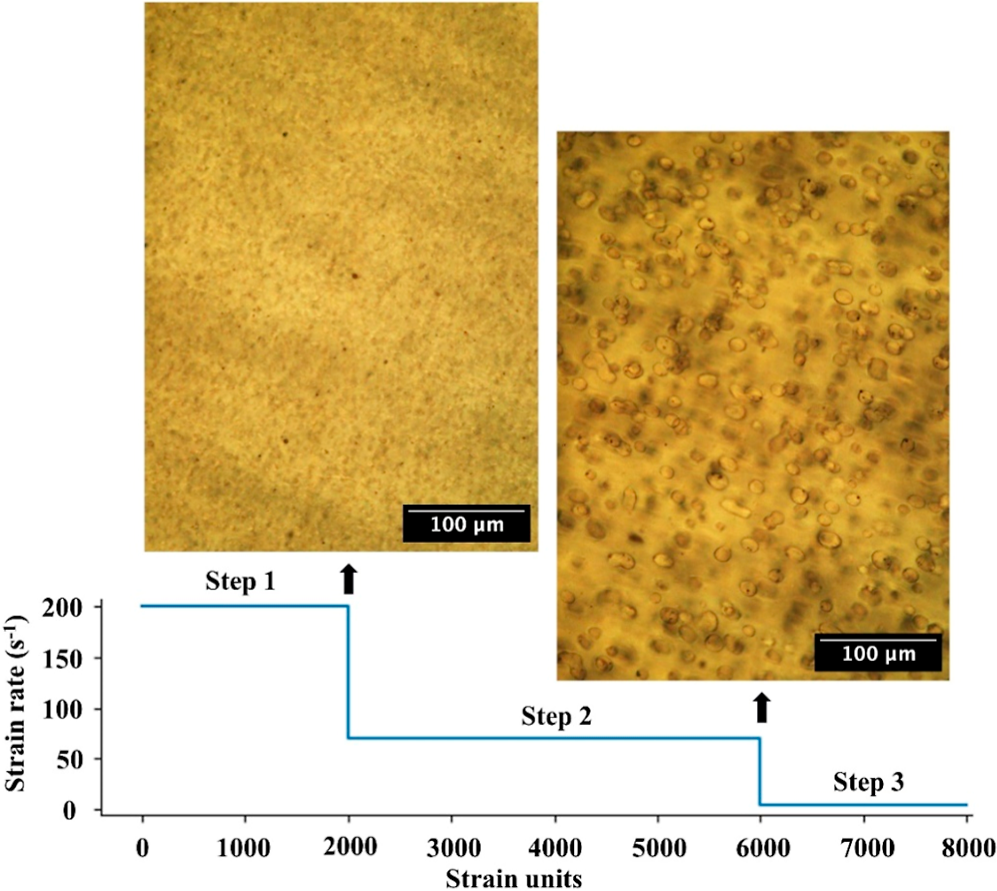
Morphologies corresponding to the preshearing of the sample at 200 s^–1^ and at 70 s^–1^. The rate in step 3 is varied, and morphological evolution is examined as a function of strain.

## 3 Results

As explained previously,^40^ the cold-mixed sample comprises solidified drops of PEO and silica particles independently suspended into the PIB continuous phase. The preshearing at 200 s^–1^ at 80 °C allows molten PEO droplets to collide with the silica particles and wet them. These particle-PEO composite drops continue colliding and coalescing. At the end of the 200 s^–1^ preshearing, the droplets of the combined phase are only a few microns in size and difficult to resolve in our images. Further shearing at 70 s^–1^ induces coalescence and growth to form droplets of roughly 10 μm in size (Figure 1). Note that these drops are more-or-less rounded, but distinctly nonspherical. Also note that some particles or the PEO drops may not have collided or coalesced with these larger composite drops or may have formed very small clusters that are not apparent at our rheomicroscopy resolution. Thus, the particle loading within each drop may deviate from the average composition. This morphology (right image in Figure 1) is designated as the initial condition for our strain-evolution study, after which shearing is continued at a rate lower than 70 s^–1^, with periodic interruptions to capture images under quiescent conditions.

### 3.1 Effect of Applied Strain Rate

We start the discussion with the case where the dispersed phase loading (ϕ_SP_ + ϕ_PEO_) is 5 vol % and the shear rate is 5 s^–1^. The sequence of images recorded during shearing is shown in the supplementary movie file and some of these images are shown in Figure 2f−j. The second row of Figure 2 shows small, irregular drops during the early stages of shearing (Figure 2f) which develop into partially coalesced aggregates as shearing proceeds. Such collision-induced growth of irregular aggregates was anticipated in the “thought experiment” in the Introduction. The aggregates appear to have a relatively large and open structure after 115 strain units (Figure 2g), but stop growing and become more compact at longer times (Figure 2i). After around 1600 strain units, many structures in the sample are found to have relaxed to an almost spherical shape.

**Figure 2:**
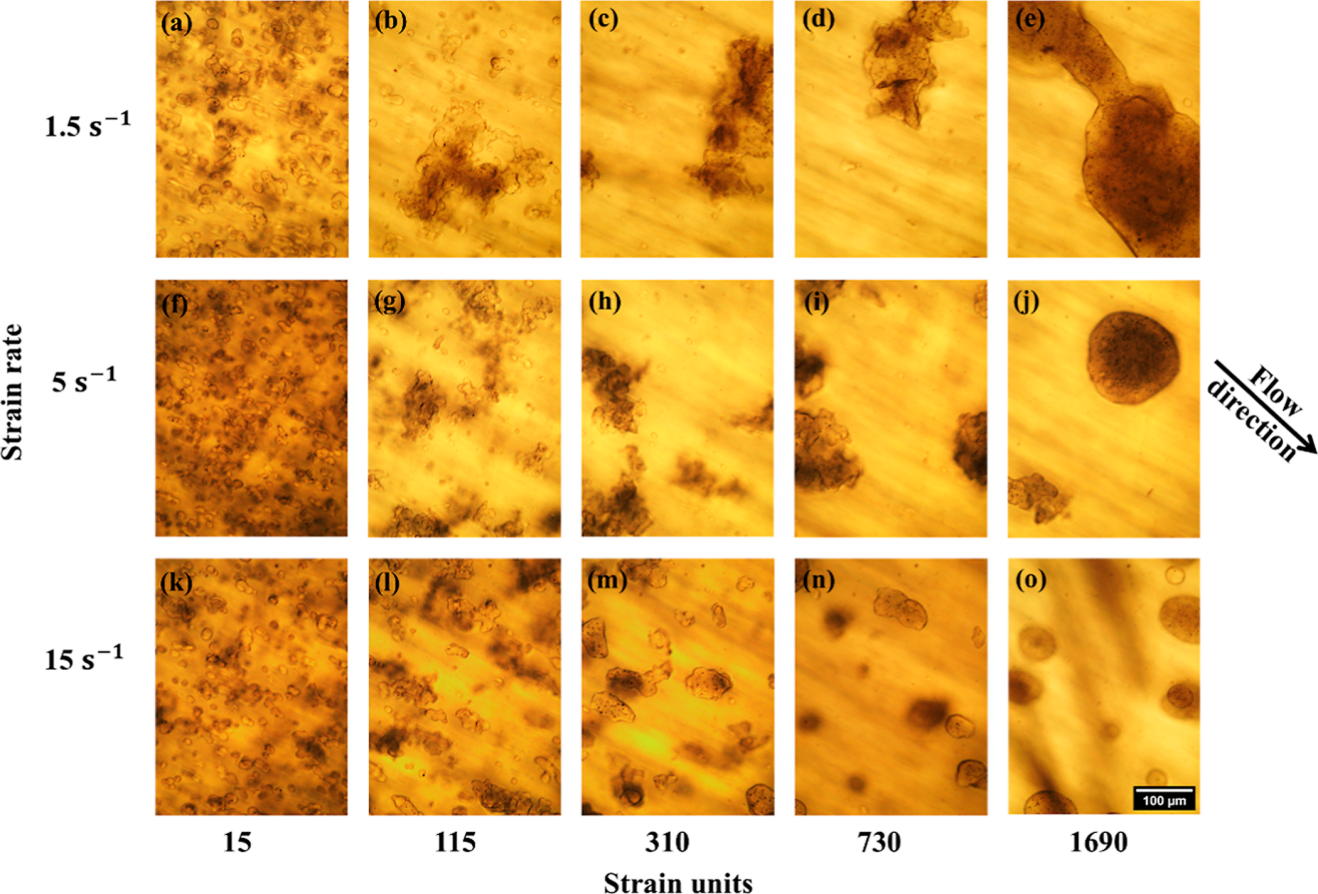
Morphological evolution during shearing and its variation across different strain rates. Scalebar at the bottom right applies to all images.

The upper and the lower rows of Figure 2 show the effect of shear rate on morphological evolution. When shear rate was increased to 15 s^–1^ (lower row), a similar evolution of morphology was observed: an initial growth into larger, irregular-shaped drops, followed by a reversion to a spherical shape under continued flow. Compared to shearing at 5 s^–1^, the reversion to spherical shape started at an earlier stage of shearing (compare, for instance Figure 2h where the aggregates are highly irregular vs Figure 2m where they are substantially compacted) and the partially coalesced drops did not grow as much. Indeed, the final size of the approximately spherical drops at 5 s^–1^ (Figure 2o) is well under the 200 μm gap size, which is much smaller in size than after shearing at 15 s^–1^ (Figure 2e).

The top row in Figure 2 shows the effect of shearing at a lower rate of 1.5 s^–1^. At early stages, the behavior is similar: large, open aggregates form due to partial coalescence after 115 strain units. However, the shear behavior at long times is different: while the open aggregates do become more compact, they never revert to being smooth and rounded even after shearing for a long time. The final morphology has large, irregular and compact structures whose lateral dimensions exceed the gap size.

The thought experiment in the Introduction listed three pathways for morphological evolution under continuous shear: growth of irregular aggregates to a size comparable to the system size (gap height in our case), deformation and reorganization of the aggregates, and a steady state where drop aggregation and breakup balance each other. Figure 2 is consistent with the first and second possibilities, and notably, the last row suggests that flow-induced deformation and reorganization of the aggregates can occur even without significant constraints from the system size. To our knowledge, this is the first report in the literature to show that partially coalesced drops may appear only transiently in flow, and further that they may survive almost indefinitely at low shear rates.

### 3.2 Effect of Volume Fraction of Dispersed Phase

Here we examined the effect of volume fraction on the subsequent compaction and reversion to sphericity. Figure 3 shows the morphological evolution of blends with total volume fraction (ϕ_SP_ + ϕ_PEO_) of dispersed phase ranging from 2% to 8% under flow at a single shear rate, 5 s^−1^. With increasing total volume fraction of dispersed phase, partially coalesced structures grow more rapidly (compare Figure 3k,f,a, in increasing order of dispersed phase fraction). This may be rationalized readily: the collision frequency at a fixed shear rate increases with volume fraction (a quadratic dependence of collision frequency on volume fraction is expected at low volume fraction when pairwise collisions dominate), and the higher collision frequency leads to rapid growth of partially coalesced aggregates. At 8 vol % dispersed phase, the structures also revert to rounded shapes over a smaller time (or strain) (compare Figure 3d,i). At a 2% volume fraction of dispersed phase, however, the long-time behavior is distinctly different. The small irregular aggregates do not approach nearly spherical shape; even after 1690 strain units, they remain highly irregular. We conclude therefore, that the reversion of drops to spherical structures becomes extremely slow at low volume fraction of the dispersed phase.

**Figure 3:**
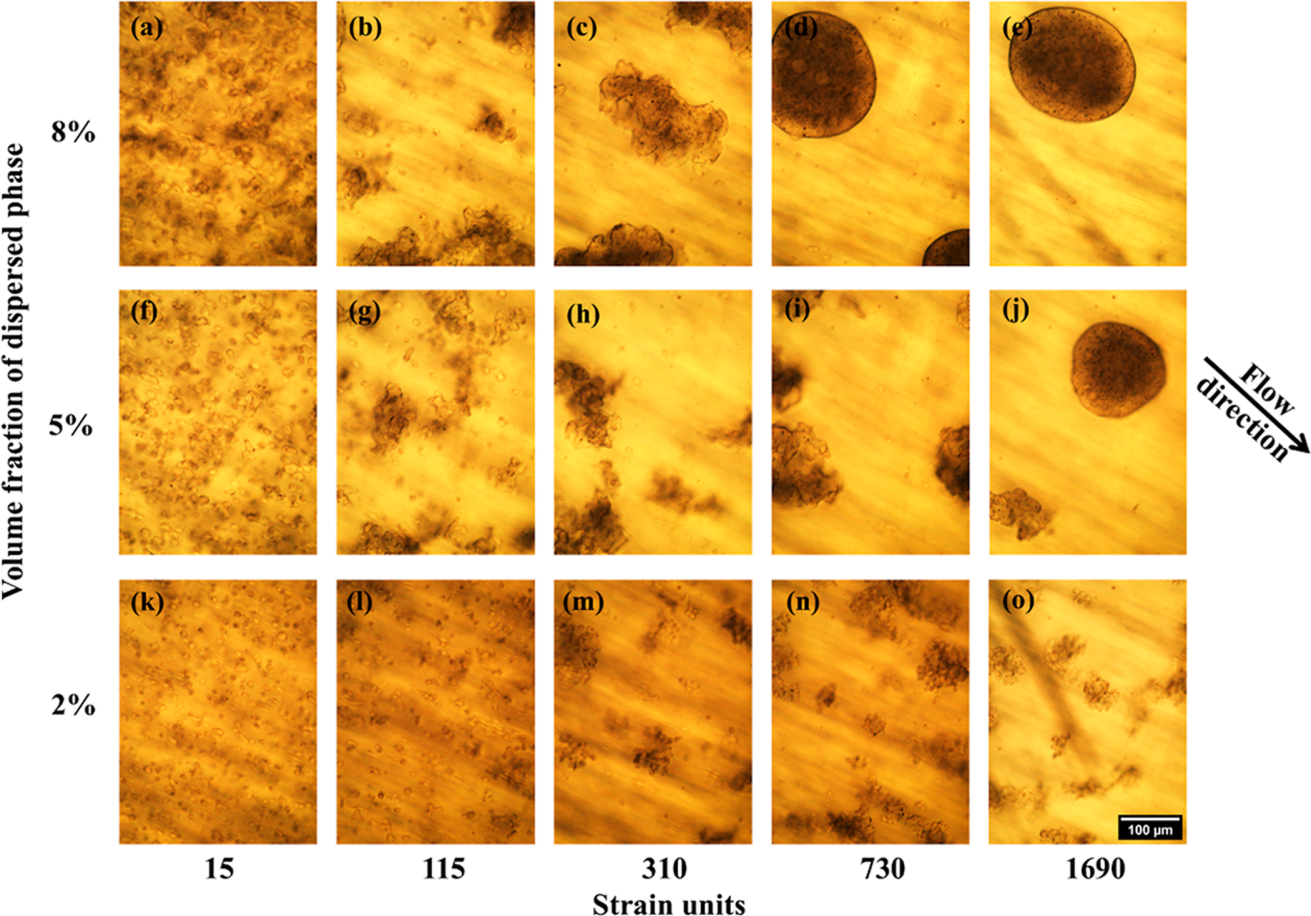
Evolution of morphology at different volume fractions. The second row of images is identical to the second row in Figure 2. Scalebar at the bottom right applies to all images.

Finally, although particle-free mixtures were not studied systematically in this research, we conducted limited experiments on PIB/PEO blends subjected to the same flow protocol as Figure 1. These experiments showed that the PEO drops were much smaller than those in the particle-containing mixtures, and further, showed relatively little coalescence when the shear rate was reduced. These results strongly suggest that particles promote coalescence. In fact, particles that are preferentially wetted by the drop phase are known to promote coalescence.^41–47^ This can be readily understood in terms of the bridging-dewetting mechanism well-known in the foams literature, see Pugh^48^ and additional citations in ref 41.

## 4 Discussion

Figure 4 summarizes the morphological evolution of ternary polymer blends in schematic form. After preshearing at a high shear rate, the highly particle-filled drops take on rounded, but not exactly spherical shapes, where the drop size increases with volume fraction of the dispersed phase. Upon subsequent shearing at a lower rate, (1) the drops undergo partial coalescence into irregular shapes, (2) these irregular drops become more compact and might eventually approach sphericity upon extended shear, (3) increasing the volume fraction or shear rate causes drops to approach sphericity at shorter times/fewer strain units, whereas at low volume fraction or shear rate, drops retain the irregular shape and do not become spherical even after several hundred strain units, (4) increasing the volume fraction or decreasing shear rate increases the size of the structures in the final morphology.

**Figure 4:**
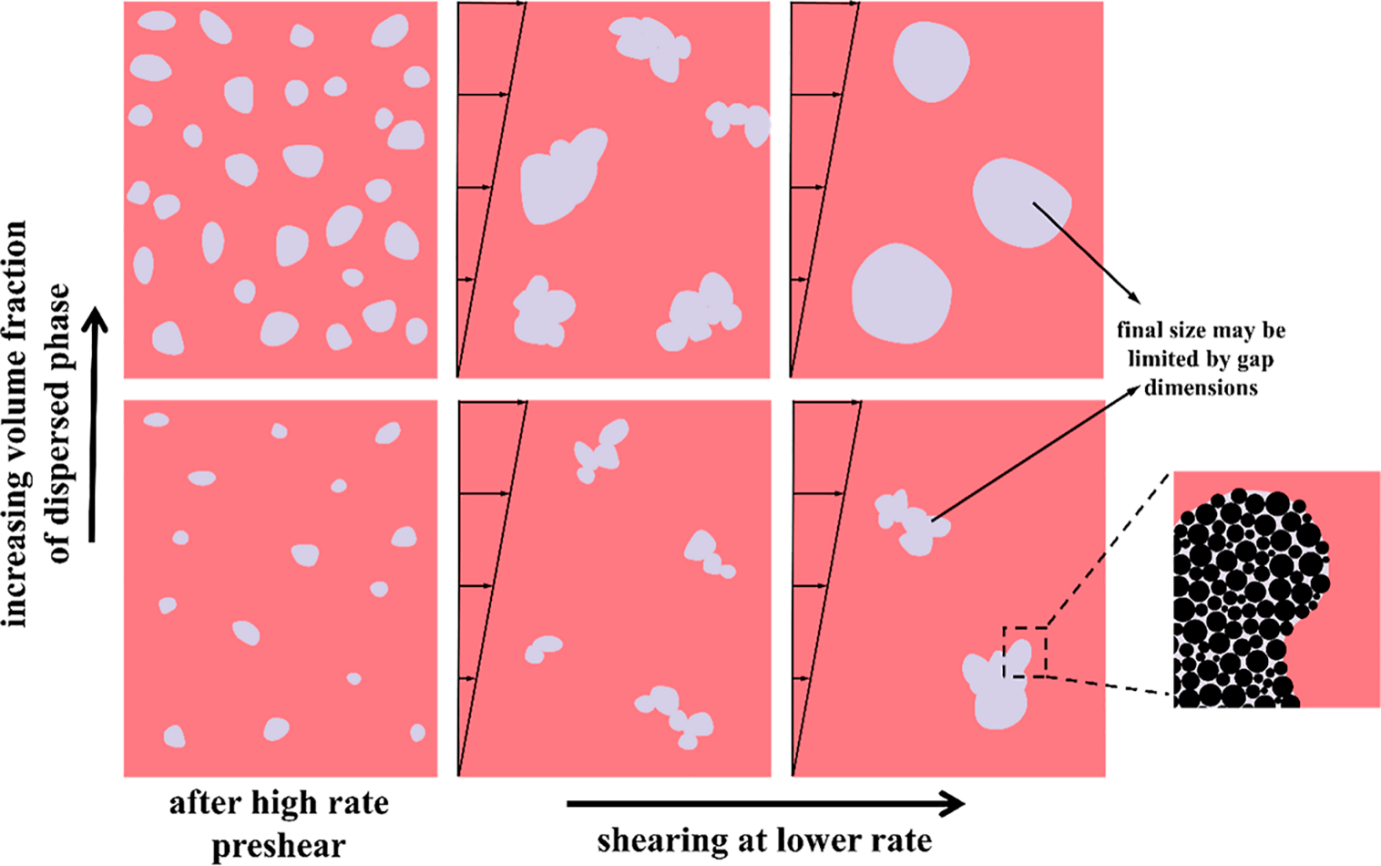
Cartoon of the effect of volume fraction of dispersed phase on morphological evolution of partially coalesced dispersed phase. The particles are shown explicitly in the magnified image on the right. Note that this magnified image shows some particles protruding out of the interface (see text).

Before proceeding with a detailed discussion of the results, we briefly discuss the location of the particles. The images clearly show that the particles are predominantly located within the drops. Yet, is it possible that some particles are at the interface? Previously we had shown that these silica particles have a strong preference to be wetted by the PEO phase rather than the PIB phase.^36,38,49^ Specifically, using scanning electron microscopy (SEM), we had examined ternary mixtures of various PEO/particle ratios, and hence various ϕ_combined_ values. At relatively low values of ϕ_combined_, the particles are entirely engulfed by the PEO. Figure S2 shows examples at ϕ_combined_ of 0.36 and 0.33, where the interface appears nearly or entirely devoid of particles, testifying to their strong preference to be wetted by PEO. However, at relatively high ϕ_combined_, (e.g., 0.62, 0.52, and 0.5 are shown in Figure S3), the particles may protrude out of the interface since there is insufficient PEO to engulf all the particles. This latter situation is likely here due to the relatively high ϕ_combined_ = 0.565. Thus, even though we cannot resolve details of the particles at the interface, we presume some particles protrude out of the interface. This may be either because the particles have preferential (but not full) wettability toward PEO, and/or because there is insufficient PEO to engulf all the particles.

We now turn to discussing the main results. Newtonian drops adopt approximately ellipsoidal shapes under steady shear flow, and upon cessation of shear, they recoil back to spherical shape due to capillarity. The fact that our drops can maintain grossly nonellipsoidal shapes under flow and nonspherical shapes under subsequent quiescent conditions suggests solid-like behavior. However, since they are capable of permanent shape changes under flow, they cannot be treated as elastic objects characterized by a modulus.^50^ Instead, it is more appropriate to think of them as “blobs” of plastic or elastoplastic material.^51^ The fact that these drops are irregular implies that the yield stress of this material must exceed the variations in capillary stress within the drops. The physical picture therefore is that shear flow drives collisions and coalescence of these blobs, but their solid-like rheology resists capillary pressure and prevents them from achieving smooth ellipsoidal shapes under flow, or perfectly spherical shapes upon cessation of flow. While the solid-like behavior of the particle-filled dispersed phase can explain the partial coalescence and irregular drop shapes, the subsequent recovery of spherical shapes under extended shearing requires understanding the role of viscous stresses. At first glance, it seems puzzling that viscous stresses induce a reversion to sphericity; in Newtonian systems, one usually expects viscous forces to deform drops *away* from sphericity. Why then do viscous stresses induce gradual reversion toward nearly spherical shapes in our experiments?

One possible explanation is that the blobs rotate around the vorticity direction due to shear flow. For a drop that is not too far from spherical, the rotational period is roughly ${\mathrm{\gamma ̇}}^{-1}$ . This induces an oscillatory tension-compression stress equal to the magnitude of the shear stress. This is illustrated in Figure 5a where the material element shown by the small gray square experiences a change in stress state as it is rotated. It is well-recognized that in the multiphase flow literature that oscillatory strain can induce microstructural rearrangements of the dispersed phase, see for example^52–58^ and citations therein. This literature includes suspensions as well as emulsions, across a wide range of dispersed phase loadings, and in colloidal as well as non-Brownian systems. This microstructural rearrangement of the dispersed phase is accompanied by “mobilization” that is evident by change in macroscopic measurements, e.g. a decrease in viscosity, onset of yielding, or facile motion of an intruder.^52,58–60^ In the current situation, we speculate that these repeated internal rearrangements due to the oscillatory stress, combined with the continuous effect of capillary pressure, eventually bring the drop to sphericity.

**Figure 5:**
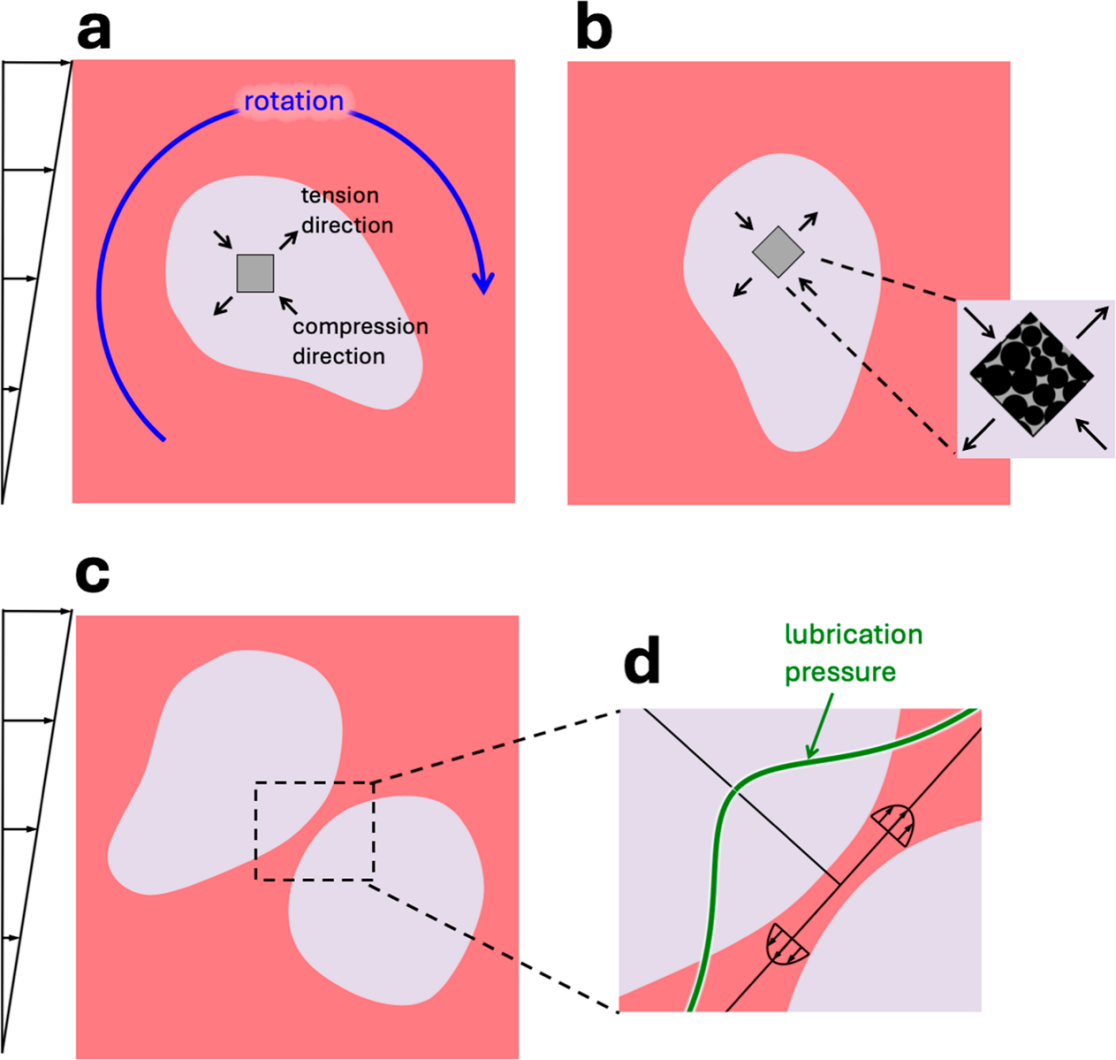
(a) A single irregular particle-filled drop in shear flow rotates due to vorticity. Any material element in the blob (shown as a gray square) experiences a periodic tension-compression due to the rotation. (b) The magnified view shows that the material element in the drop phase is crowded with particles. These particles may rearrange due to tension-compression forces as the material element rotates. (c) Collision of two blobs in shear flow. (d) Magnified view of near-contact region to show the lubrication flow in the continuous phase. The thick green curve shows the local lubrication pressure which can far exceed the mean shear stress applied. In all images, particles inside the blob are not shown for clarity.

Yet, the vorticity-based physical picture of Figure 5a cannot explain the effect of the dispersed phase loading on the morphological evolution. This is because all three dispersed phase loadings (with (ϕ_SP_ + ϕ_PEO_) values of 2%, 5%, and 8%) are relatively dilute. Accordingly, the shear stress during continuous shear is expected to be approximately $\mathrm{\gamma ̇}\times \eta_{m}$ at all volume fractions, as is indeed observed experimentally (Supporting Information S2). Since all three cases are expected to have a similar magnitude of the oscillatory tension-compression stress at a particular shear rate, the physical picture of the previous paragraph would suggest similar morphological evolution at all three dispersed phase loadings. Contradicting this expectation, Figure 3 shows that high dispersed phase volume fractions allow large and rapid shape recovery during flow, whereas low volume fractions allow highly irregular shapes to persist for the entire duration of shearing. This dependence on the dispersed phase volume fraction suggests that the micromechanics of a single nonspherical drop cannot entirely explain the results; interactions between the blobs also play a role.

We now propose an analogy to the industrial process of *spherical agglomeration* in which a suspension of fine particles is forced to agglomerate into spherical “beads” by adding a wetting fluid and agitating the blend in a shaker or tumbler.^61^ To illustrate the analogy, we conducted a separate experiment using cornstarch particles and water as the wetting fluid. The water/cornstarch weight ratio was empirically adjusted to be 0.416 which was found to be suitable for this illustration. The water-cornstarch blend was first shaken vigorously in a polypropylene container to distribute the water evenly in the starch particles, followed by tumbling the container at 100 rpm for 300 rotations. The container was kept closed throughout to prevent evaporation. This resulted in irregular polydisperse aggregates (Figure 6a). The tumbling speed was then reduced to 15 rpm, and after 20 rotations (Figure 6b), well-defined aggregates of slightly irregular shape appeared. Continued tumbling at 15 rpm for an additional 300 rotations allowed the aggregates to grow, but crucially they did not become more irregular, as would happen if the aggregates from Figure 6b merely merged together. Instead, their surface became much smoother and rounded. The underlying reason, well-accepted in the literature on spherical agglomeration, is that low level of agitation causes repeated collisions and merging of aggregates, but the collisions also smoothen the edges and “reshape” them into smooth, approximately spherical shapes (Figure 6c,f).

**Figure 6:**
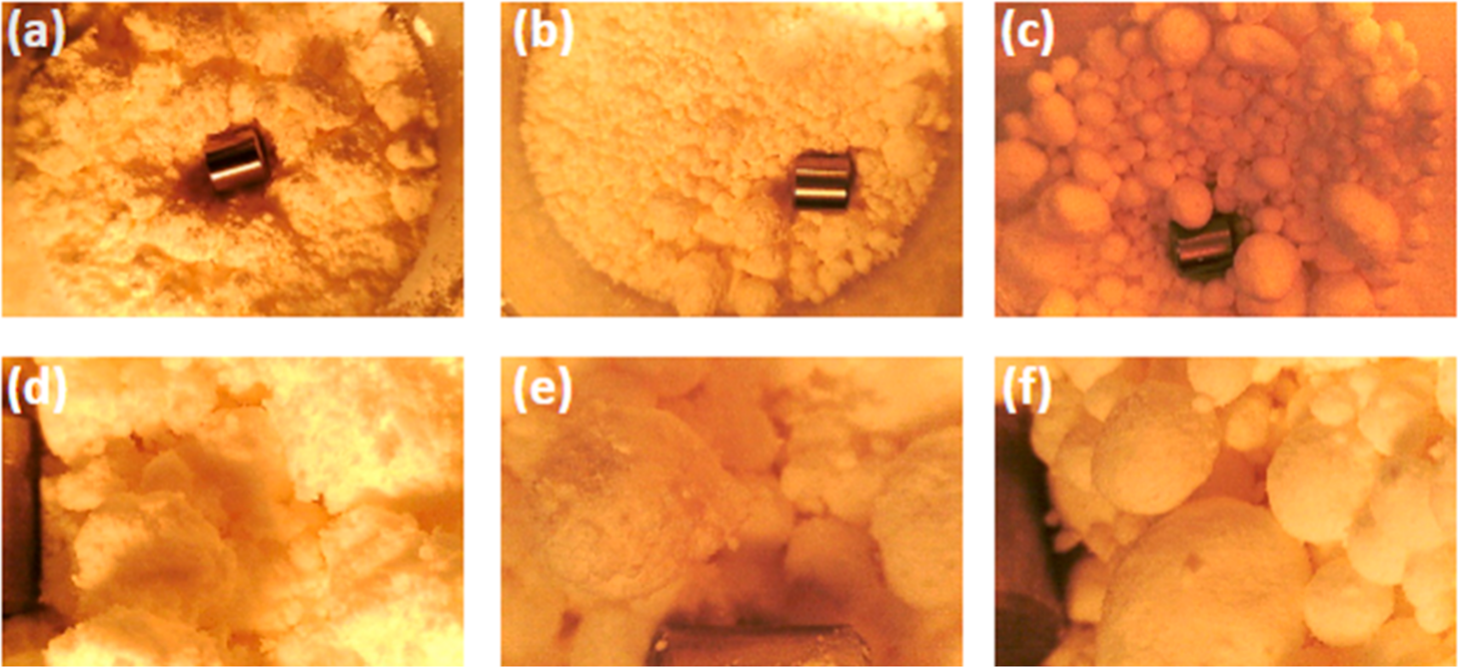
Experiments with water/cornstarch mixtures. (a,d) After tumbling the container at 100 rpm for 300 rotations, (b,e) after continued tumbling at 15 rpm for 20 rotations, and (c,f) after continued tumbling at 15 rpm for 300 rotations. Lower row images are the same samples as upper row, but at higher magnification. A metal cylinder of 0.25 in. length and 0.25 in. diameter is placed in the container as a size scale.

Analogously, we propose that reversion to sphericity in our experiments is driven by repeated collisions between blobs. We acknowledge, of course, that the collision between two water-cornstarch aggregates is very different from that between the particle-filled drops in Figure 3 due to the viscous continuous phase in the latter case. The physics of such viscous collisions has been well explored by experiments on shear-induced collision of spherical particles or drops.^62–64^ As the colliding objects approach each other (Figure 5b), the thin film of intervening fluid starts draining. This drainage is sustained by a pressure gradient in the draining film illustrated by the thick green line in Figure 5b. The last stages of drainage are typically promoted by attractive forces between the colliding objects. If the entire drainage process can be completed within the time of collision (of the order of ${\mathrm{\gamma ̇}}^{-1}$ ), the colliding objects can touch. In the present case, where the colliding objects are particle-filled drops, the two individual drops may merge together to create a single, partially coalesced drop. If, however, the drainage is too slow, the objects approach closely without touching, rotate around each other, and separate again.^62–64^ However, in this latter case, the near-contact region can experience lubrication pressures that far exceed the overall shear stress. To understand this, consider two spheres of radius *R* approaching each other at a velocity *v*. When their separation *h* is small (i.e., *h* ≪ *R*), the peak lubrication pressure in the near-contact region^65,66^ is 3*Rv*η_m_/*h* ^2^. In simple shear, we may set *v* = γ̇R, which is the typical approach velocity for two objects of radius R. Accordingly, the peak lubrication pressure is expected to be $\left[3R^{2}/h^{2}\right]\times \left(\mathrm{\gamma ̇}\eta_{m}\right)$ . Here the quantity $\mathrm{\gamma ̇}\eta_{m}$ in the round brackets is the typical value of the steady shear stress and the tension-compression oscillatory stress. If the drops are in close proximity, the quantity in the square brackets may be over 100, suggesting that the peak lubrication pressure is far higher than the mean stress level. A calculation can illustrate the actual values expected: for drops of radius *R* = 50 μm, at a shear rate of 5 s^–1^ and η_m_ = 10 Pa.s, the typical shear stress level $\mathrm{\gamma ̇}\eta_{m}$ is 50 Pa. At an instant when the separation is 10 μm (which is still far larger than the particle size) the quantity in the square brackets is 75, and hence the peak lubrication pressure is 3750 Pa. Thus, it is readily possible that the mean level of viscous stress may not be able to distort drops on a gross level, but the local lubrication pressure may distort the drops locally in the collision zone. We acknowledge that the calculation is strictly valid only for spheres in a head-on collisions, not for irregular drops colliding in shear flow. Nevertheless, qualitatively it makes the case that local stresses due to collisions between the particle-filled drops can far exceed the stress experienced by a single isolated drop in the same flow. These high local stresses in the near-contact region may drive particle rearrangements far more effectively than the oscillatory tension-compression stress from Figure 5a. In summary, repeated collisions between nonspherical drops may be responsible for the relatively rapid shape changes at higher drop fractions; this is analogous to spherical agglomeration except that the collisions are viscous.

We emphasize that mechanisms of Figure 5a vs Figure 5b are not mutually exclusive, and both may be relevant. Yet, the effect of volume fraction on morphology evolution supports a significant role of the collision picture of Figure 5b. Incidentally, note that both mechanisms become inactive if the nonspherical drops become comparable in dimension to the gap; Figure 5a because the narrow gap would suppress rotation around the vorticity direction, and Figure 5b because all blobs would move at approximately the center-line velocity in the gap, and collisions would become infrequent. Finally, we emphasize that as per Figure 5, the reversion to sphericity is primarily driven by viscous forces, not by capillarity. In fact capillary forces alone (i.e., under quiescent conditions) do not force drops to revert to sphericity.

Both mechanisms of Figure 5 are based on the idea that viscous stress, either oscillatory tension-compression or due to collisions, induces yielding. Therefore, it is natural to ask: what is rheology of the particles-in-PEO suspension which comprises the drops? Unfortunately we were unsuccessful in quantifying the rheology. We attempted small- and large-amplitude oscillatory experiments, steady shear experiments at controlled shear rate, and creep experiments at controlled stress. Experiments were conducted in a parallel plate rheometer with and without roughened plates, at various gaps, and different flow histories.^67^ These experiments gave irreproducible measurements with storage modulus values that varied by up to an order of magnitude. Squeeze flow experiments were also attempted, but the results could not be fitted to simple models of yielding fluids. We believe that the relatively high volume fraction of the particles are the chief reason for this difficulty; indeed the PEO + silica sample appears “dry” when mixing, is difficult to mix homogeneously, and is even difficult to load into the rheometer.

Finally, the above discussion presumes that the resistance to shape change comes from the solid-like behavior within the drops. Yet, as discussed at the beginning of Section 4, we cannot entirely rule out interfacial adsorption. Interfacial adsorption may endow the interface with some solid-like behavior and this may contribute to the nonspherical drop shapes. Yet, all of the discussion above, including the two mechanisms of Figure 5, applies even if interfacial adsorption contributes to irregular drop shapes. In that case, particle rearrangement due to drop collisions would include not just rearrangement in the bulk of the drops, but also rearrangement on the surface and desorption from the surface into the bulk PEO phase.

## 5 Conclusion

To summarize, we have conducted a flow-visualization study of the morphological evolution of dilute emulsions of particle-containing-drops subjected to a stepdown in shear flow rate. The drops adopt irregular shapes after the initial high-rate shear, followed by coalescence at lower rate. The particle-containing drops coalesce to a greater extent as compared to particle-free drops, i.e. the particles promote coalescence. However, the particle-containing drops fuse, but do not immediately revert to a spherical shape, a phenomenon known as partial coalescence. The central experimental result of this paper is that extended shearing at a low rate causes gradual reversion of the partially coalesced drops into rounded or nearly spherical shapes. The reversion to sphericity is found to be very slow at low shear rates and/or at low loadings of the dispersed phase. Thus, partial coalescence and the formation of irregular structures in flow is a transient phenomenon associated with a sharp decrease in flow strength. The presence of nonspherical shapes is readily attributable to the solid-like behavior of the drops which comes from the high particle fraction inside the drops, and possibly to particles also present at the interface. The reversion to sphericity cannot be attributed to interfacial tension since the drops can sustain nonspherical shapes indefinitely under quiescent conditions. Instead, we propose that the chief mechanism is that when irregularly shaped drops collide, the viscous stress in their near-contact region induces localized yielding and particle rearrangements. Thus, repeated drop collisions gradually smoothen the drops toward sphericity.

These morphological observations highlight the importance of processing history in formulation engineering. If irregularly shaped drops are desired, e.g. to stabilize multiphase emulsions in the food industry, overmixing may be undesirable. A particularly interesting implication of these results is that they suggest a pathway to creating cocontinuous morphologies: first shear at high rate to create a dispersion of particle-filled drops, and then shear briefly at low rate to partially coalesce them into volume spanning, i.e. percolating structures. Once again, overshearing at the second stage may be undesirable.

The research here was conducted with polydisperse spherical particles which do not have strong interactions when suspended in PEO. However, particles used in practical applications are often much more complex. If the dispersed phase is filled with particles such as fumed silica, carbon nanotubes, or platelets, solid-like rheology may develop at much lower particle loadings. Moreover, gradual aggregation of such particles can also change the solid-like behavior over time, a phenomenon especially recognized as the thixotropic behavior of fumed silica suspensions.^68^ The needle-like fat crystals examined in previous research^16,23^ may also break under flow, therefore irreversibly changing the solid-like behavior of the drop phase upon shearing. Even more severe morphological changes may be expected in such cases.

## Supplementary Material





## Notes

Morphological evolution of 5% dispersed phase at shear rate of 5 s^–1^ (MP4)
Information on silica particles, SEM images of various blends, viscosity data from the step-down experiments (PDF)
