# Supplementary material for: Particle-Filled Emulsion Drops Show Flow-Induced Partial Coalescence, but Only Transiently
Source: Ind Eng Chem Res. 2025 Nov 10;64(46):22185–95. doi: 10.1021/acs.iecr.5c02170 (PMC12636018; doi:10.1021/acs.iecr.5c02170)
Supplement: Supplementary file 2 [file ie5c02170_si_002.pdf]

**Supporting Information**

# Particle-filled emulsion drops show flow-induced partial coalescence, but only transiently.

Jovina Vaswani[1], Sachin Velankar* [1,2]

*[1]Department of Chemical Engineering, University of Pittsburgh, Pittsburgh, Pennsylvania 15261, USA*
*[2]Department of Mechanical Engineering and Material Science, University of Pittsburgh, Pittsburgh, Pennsylvania 15261, USA*

**Corresponding author; e-mail address: velankar@pitt.edu*

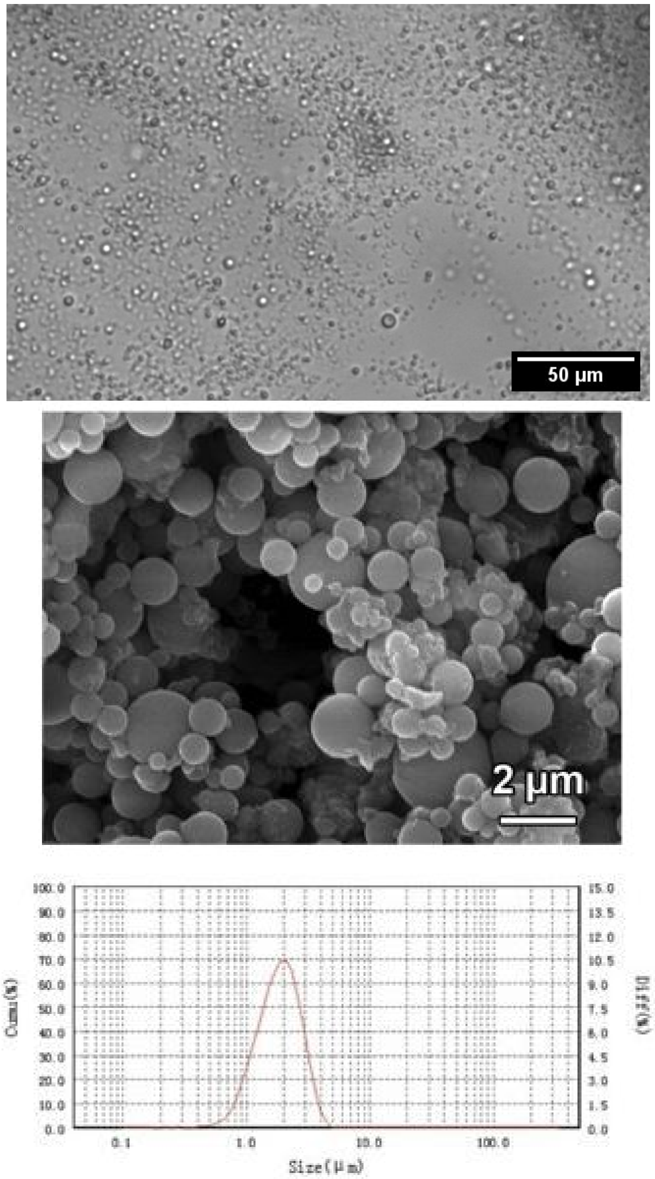


Figure S1: Top: Optical microscopy image of silica particles in PDMS. Middle: Electron microscopy image reproduced from T. Domenech and S. S. Velankar, *"Capillary-driven percolating networks in ternary blends of immiscible polymers and silica particles," Rheologica Acta, 2014*. Bottom: Particle size distribution provided by the vendor (http://industrialpowder.com/index.php?c=117&p=133)

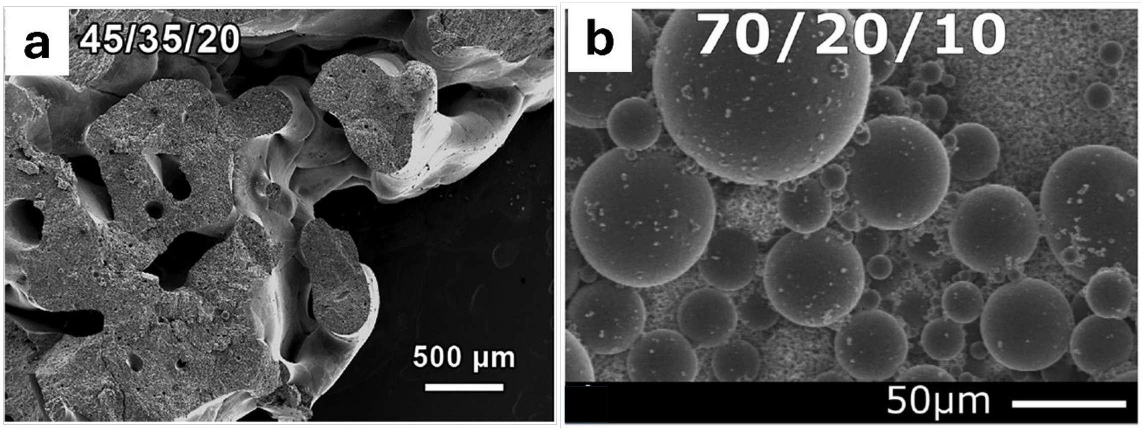


Figure S2: SEM images of mixtures with composition PIB/PEO/silica as noted at the top of each image. Unlike Fig. S3, these compositions have a relatively high PEO:silica ratio. In both cases, the PIB phase was extracted leaving behind the particles and the PEO. (a) has a bicontinuous microstructure where particles are visible inside the PEO phase (image taken from Domenech, Trystan and Sachin S. Velankar, "*Microstructure, Phase Inversion and Yielding in Immiscible Polymer Blends with Selectively Wetting Silica Particles*.", *Journal of Rheology, 2017*. (b) has a droplet-matrix structure and therefore "falls apart" and is imaged on a filter paper. The frozen PEO drops, which contain the particles, are visible. In both cases, the interface remains smooth suggesting that the particles are fully-wetted by the PEO and do not adsorb at the interface (image taken from J. Y. Yang, David Roell, Martin Echavarria, Sachin S. Velankar, "*A microstructure-composition map of a ternary liquid/liquid/particle system with partially-wetting particles", Soft Matter, 2017).*

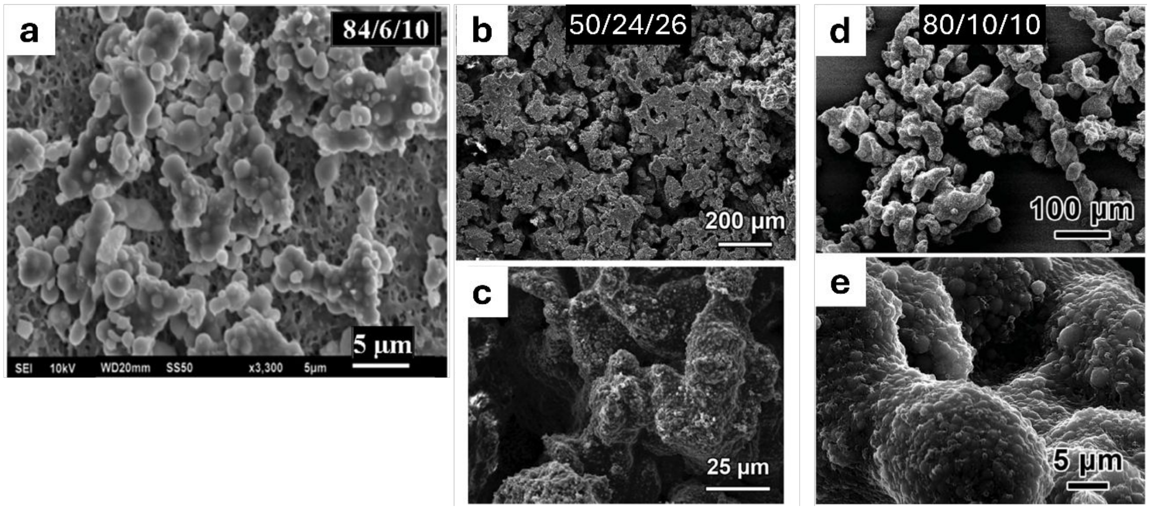


Figure S3: SEM images of mixtures with composition PIB/PEO/silica as noted at the top of each image. Unlike Fig. S2, in all cases, these compositions have a relatively low PEO:silica ratio which increases from left to right. (c) is a magnified view of (b) (images taken from Domenech, T., J. Y. Yang, S. Heidlebaugh, and S. S. Velankar, "*Three Distinct Open-Pore Morphologies from a Single Particle-Filled Polymer Blend.*", *Physical Chemistry Chemical Physics, 2016*), whereas (e) is a magnified view of (d). The PIB phase was extracted leaving behind the particles and the PEO (images taken from T. Domenech and S. S. Velankar, "*On the rheology of pendular gels and morphological developments in paste-like ternary systems based on capillary attraction*.", *Soft Matter, 2015*. In (a (taken from Derrick Amoabeng, David Roell, Kendal M. Clouse, Brian A. Young, Sachin S. Velankar, "*A composition-morphology map*

*for particle-filled blends of immiscible thermoplastic polymers", Polymer, 2017*) and (d&e), due to the high PIB fraction, the combined PEO+silica phase forms the dispersed phase which is deposited on a filter paper for imaging. In contrast, (b&c) has a bicontinuous microstructure where particles are visible inside the PEO phase. Note how with increasing PEO:silica ratio, the particles are increasingly engulfed by the PEO. In (d&e), the particles protrude out of the interface slightly.

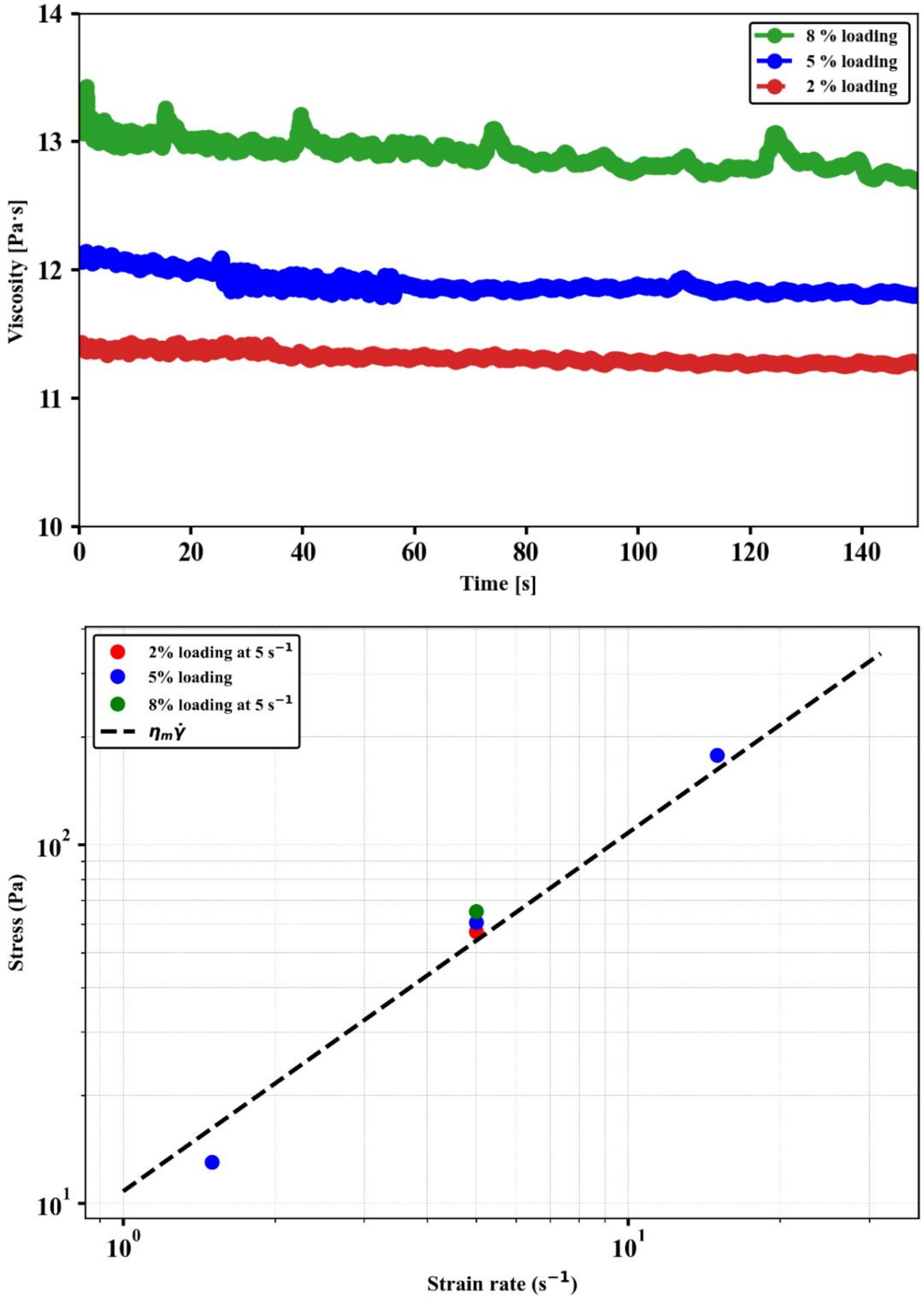


Figure S4: Top: Example of viscosity vs time after stepping down the shear rate from 70 s$^{-1}$ to 5 s$^{-1}$. Note that the viscosity (and hence shear stress) changes only modestly with total dispersed phase loading, presumably because all these samples are relatively dilute. Bottom: The shear stress measured at long times after stepping down the shear rate to three different values. The three points at the rate of 5 s$^{-1}$ correspond to the average stress at late stages from the upper plot.
